# State- and frequency-dependence in autonomic rebalance mediated by intradermal auricular electroacupuncture stimulation

**DOI:** 10.3389/fnins.2024.1367266

**Published:** 2024-05-23

**Authors:** Sen Yang, Yu-Rui Wu, Zheng Zhan, Yan-Hong Pan, Jin-Feng Jiang

**Affiliations:** Key Laboratory of Acupuncture and Medicine Research of Ministry of Education, Nanjing University of Chinese Medicine, Nanjing, China

**Keywords:** vagus nerve stimulation, autonomic nervous system, frequency-dependency, ANS imbalance, HRV

## Abstract

**Background:**

Vagus nerve stimulation (VNS) improves diseases such as refractory epilepsy and treatment-resistant depression, likely by rebalancing the autonomic nervous system (ANS). Intradermal auricular electro-acupuncture stimulation (iaES) produces similar effects. The aim of this study was to determine the effects of different iaES frequencies on the parasympathetic and sympathetic divisions in different states of ANS imbalance.

**Methods:**

We measured heart rate variability (HRV) and heart rate (HR) of non-modeled (normal) rats with the treatment of various frequencies to determine the optimal iaES frequency. The optimized iaES frequency was then applied to ANS imbalance model rats to elucidate its effects.

**Results:**

30 Hz and 100 Hz iaES clearly affected HRV and HR in normal rats. 30 Hz iaES increased HRV, and decreased HR. 100 Hz iaES decreased HRV, and increased HR. In sympathetic excited state rats, 30 Hz iaES increased HRV. 100 Hz iaES increased HRV, and decreased HR. In parasympathetic excited state rats, 30 Hz and 100 Hz iaES decreased HRV. In sympathetic inhibited state rats, 30 Hz iaES decreased HRV, while 100 Hz iaES decreased HR. In parasympathetic inhibited rats, 30 Hz iaES decreased HR and 100 Hz iaES increased HRV.

**Conclusion:**

30 Hz and 100 Hz iaES contribute to ANS rebalance by increasing vagal and sympathetic activity with different amplifications. The 30 Hz iaES exhibited positive effects in all the imbalanced states. 100 Hz iaES suppressed the sympathetic arm in sympathetic excitation and sympathetic/parasympathetic inhibition and suppressed the vagal arm and promoted the sympathetic arm in parasympathetic excitation and normal states.

## Introduction

The balance between the sympathetic and parasympathetic nervous systems is critical for maintaining homeostasis. A persistent autonomic nervous system (ANS) imbalance state is a preceding factor for many disorders including chronic inflammatory disorders (Kaniusas et al., 2019a; Bellocchi et al., 2022). A primary abnormality of the ANS or its dysregulation can cause ANS imbalance, which could occur in isolation or as manifestations of other disease states (Hyam et al., 2012; Goldstein, 2021). ANS imbalance is a prominent feature in pathological conditions (Barthelemy et al., 2022) including chronic heart failure (De Ferrari et al., 2011; Libbus et al., 2022), epilepsy (Akyuz et al., 2021; Hodl et al., 2021), immune-mediated inflammatory diseases (Sallam et al., 2018), psychiatric disorders (Thompson et al., 2021; Austelle et al., 2022; Tan et al., 2022), and long COVID-19 (Badran et al., 2022b; Jammoul et al., 2022; Malkova and Shoenfeld, 2023). The ANS is therefore an attractive target for the treatment of such diseases (Bonaz et al., 2017). ANS rebalancing strategies are therefore of great clinical interest (Saavedra-Alvarez et al., 2022).

Vagus nerve stimulation (VNS) presents a novel concept of direct ANS regulation via the vagal circuits (Balasubramanian et al., 2017) in addition to the anti-epileptic and anti-depression effects approved for therapeutic use by the US FDA. VNS effectively alleviates ANS dysfunction-associated symptoms in its indications such as inflammatory bowel diseases, rheumatoid arthritis, obesity, and pain (Bonaz et al., 2017). However, VNS requires costly and invasive electrode implantation, with associated side effects (Ben-Menachem et al., 2015).

The auricular concha is densely innervated by free nerve endings of the vagus nerve (Hilz, 2022). The auricular branch of the vagus nerve (ABVN), which is a unique somatic branch of the vagus nerve (He et al., 2013; Bermejo et al., 2017), can be activated by stimulation of the auricular concha area (Butt et al., 2020). The area of intervention in the rat in this study corresponds to the human auricular concha, which is mostly innervated by the vagus nerve, distinguishing it from the innervated sections of the great auricular nerve and the auriculotemporal nerve (Gao et al., 2008). Transcutaneous auricular vagal nerve stimulation (taVNS) produced effects similar to those of VNS in animal experiments and pre-clinic trials (Badran and Austelle, 2022; Baig et al., 2022). Stimulation of the ABVN via the cymba concha activates the nucleus of the solitarius tract, the locus coeruleus (LC), and other primary and higher-order vagal projections in the brainstem and forebrain (Mercante et al., 2018). taVNS affects sympathetic nuclei such as the LC on the autonomic feedback pathway via the nucleus of the solitary tract (NST) (Rosso et al., 2020; Dolphin et al., 2022). Therefore, ABVN stimulation could activate the vagal circuit as well as the sympathetic system. Thus, iaES could be a strategy to directly modulate ANS.

Intradermal auricular electro-acupuncture stimulation (iaES), an electro-acupuncture (EA) technique specialized to the auricular concha, is an innovative and convenient non-invasive therapy, developed from traditional acupuncture manipulation based on the anatomical distribution of the ABVN (Mercante et al., 2018; Butt et al., 2020). The needles in iaES remain parallel penetrated within the skin layer (Wang et al., 2021), avoiding contact with the cartilage, thereby effectively triggering vagal activation without any discomfort or pain (Yang et al., 2021). In addition, shallow needling enlarges spatial summation by transforming point stimulation into line stimulation (Wang et al., 2021). iaES effectively and safely activates the ABVN (Chen et al., 2018; Shen and Jiang, 2019) and been successfully applied in clinical studies (Liu X. R. et al., 2020; Miao et al., 2020; Wang et al., 2021). iaES directly stimulates the ABVN to reduce glycemic load (Liu X. R. et al., 2020), and shows anti-depressive effects (Wang et al., 2021), enhances gastrointestinal motility (Yang et al., 2021), and improves upper limb motor function in post-stroke hemiplegia (Miao et al., 2020).

Different VNS frequencies cause changes in neural activation patterns (Dolphin et al., 2022; Szabo et al., 2022); 300 Hz VNS caused significant hypoperfusion in the left frontal lobe and the right parietal lobe whereas 30 Hz VNS caused no significant changes in those regions (Martle et al., 2014). Frequency is also an important factor in EA. Low-frequency (1 Hz) EA suppressed inflammation via sympathetic post-ganglionic neurons, while high-frequency (120 Hz) EA-mediated suppression involves the sympathoadrenal medullary axis (Kim et al., 2008). We therefore hypothesized that different frequencies of iaES would exert differential regulatory effects on the ANS. Liu S. et al. (2020) recently described how electroacupuncture activates district autonomic networks to control inflammation in a disease state-dependent manner. Therefore, we hypothesized that state-dependency in iaES would be a distinct feature in normal and autonomic imbalance models. HRV and HR are biomarkers of ANS activity (Wolf et al., 2021). We used heart rate variability (HRV) monitoring (Michael et al., 2017; Capilupi et al., 2020) to determine the effects of different iaES frequencies in normal and ANS imbalance model rats.

## Materials and methods

### Animals

Male Sprague–Dawley (SD) rats (*n* = 120, 220–250 g) were provided by the Zhejiang Academy of Medical Sciences [experimental animal quality certificate no. SCXK (Zhejiang) 2019-002 and SCXK (Zhejiang) 2020-002]. The rats were housed in an SPF grade laboratory room at the Animal Experimental Center, Nanjing University of Chinese Medicine, maintained at 23 ± 1°C and 55% ± 5% humidity, with 12 h light illumination, and free access to water and food. All rats were acclimated to the environment for one week before the procedures. Experiments were carried out from week 2 to week 8 and were approved by the Ethics Committee of the Animal Center of the Nanjing University of Chinese Medicine (Ethics Approval No. 201910A035). The treatment and disposal of the animals were in compliance with the relevant provisions of the *Guiding Opinions on the Humane Treatment of Laboratory Animals* issued by the Ministry of Science and Technology of the People’s Republic of China.

### Generation of ANS imbalance models

Four ANS imbalance models were produced by intraperitoneal injection once a day of the corresponding chemical agents as follows: sympathetic excited state rats: 0.01% clenbuterol (0.5 mg/kg) for 20 days (Mohamed-Ali et al., 2001; Ni et al., 2006), parasympathetic excited state rats: 20% acetylcholine (0.1 mg/kg) for 7 days (Garcia-Pedraza et al., 2020), sympathetic inhibited state rats: 0.2% propranolol (10 mg/kg) for 7 days (Henriquez et al., 2018), and parasympathetic inhibited state rats: 0.1% atropine (4 mg/kg) for 7 days (Grippo et al., 2018). The last injection was administered one hour before the electrocardiogram was connected.

### Experimental animal groups and interventions

Rats were anesthetized with 20% urethane (0.5 ml/100 g) owing to its weak inhibition of the ANS (Zhou and Qu, 2008), and the subcutaneous electrocardiograph electrodes were linked to animals’ limbs. The electrocardiogram (ECG) signal lead was introduced into the biological signal acquisition and analysis system (Powerlab, AD Instruments, Colorado Springs, CO, Australia), and the heart rate (HR) and HRV were continuously recorded during the experiments. The animals’ temperature was maintained at 37 ± 1°C by feedback-controlled electric blankets throughout the experiments.

**FIGURE 1:**
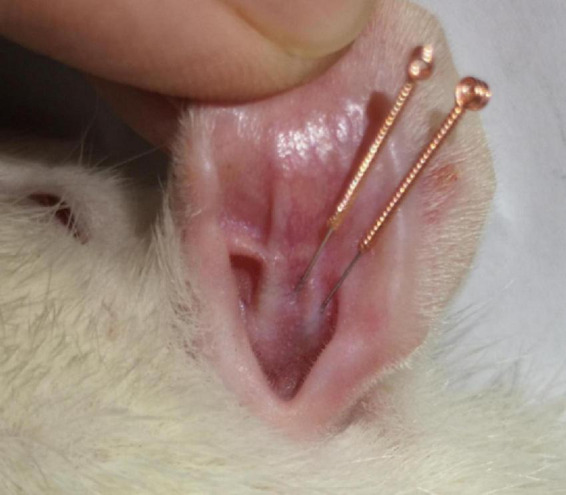
Schematic illustration of iaES.

Rats were kept supine, and two 0.18 mm × 10 mm *Huangdi* brand sterile disposable needles (Jiangsu Zhenjiang, China) were inserted parallelly into the cymba concha and cavum concha of the right ear (Figure 1; Yang et al., 2021) 6 mm of the needle was pierced through the skin, fixed with adhesive tape, and then connected to the Han’s EA apparatus (Nanjing Jisheng Technology Co., Ltd.). The stimulation parameters were: continuous wave, intensity: 2 mA, wave width: 0.2 ms ± 30%, 30 min.

**FIGURE 2:**
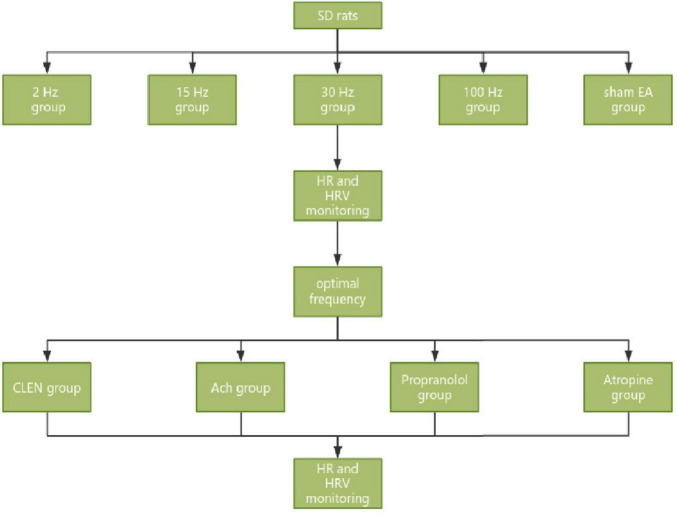
Experimental process of iaES frequency optimization in SD rats and iaES with optimized frequency in model rats with ANS imbalance.

The following experiments were conducted to optimize the effective frequency of iaES to impact HRV in rats and to assess its effects on rats with autonomic imbalance. Experimental protocols are shown in Figure 2.

Optimization of iaES frequency to modulate HRV in normal rats

40 male SD rats were sorted according to body weight and randomly assigned to five groups: 2 Hz, 15 Hz, 30 Hz, 100 Hz, and sham EA groups (*n* = 8), according to the random number table method, and received iaES intervention at the above-mentioned frequencies. Most studies have used a frequency between 20 and 30 Hz (Thompson et al., 2021). Sclocco et al. (2020) evaluated the response of brainstem fMRI to the respiratory gating taVNS (cymbal conch) at four different stimulus frequencies (2, 10, 25, and 100 Hz) (Sclocco et al., 2020). The limits on the instrument used (HANS—2000) were 15 Hz and 30 Hz. We therefore selected 15 Hz and 30 Hz for our experiments. HRV was recorded in the pre-iaES, iaES, and post-iaES phases. In the sham EA group, the needle was fixed with adhesive tape on the surface, and no electrical stimulation was added. As shown in the experimental flow chart (Figure 2), after 40 min of anesthesia adaptation, the HRV was maintained for 5 min as a baseline before iaES (30 min) and was monitored until 30 min after the intervention.

Effects of optimized iaES on HRV in rats with ANS imbalance

After adaptive feeding for one week, 80 SD rats were sorted according to body weight and randomly divided into a control group (*n* = 16) and four ANS imbalance model groups (*n* = 16) by using the random number table method, and further divided into 30 Hz iaES (*n* = 8) and 100 Hz iaES (*n* = 8) subgroups in each group. The experimental scheme is shown in Figure 2.

### HRV and HR monitoring

**FIGURE 3:**
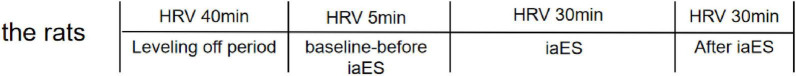
Schematic representation of iaES HRV and HR monitoring.

The HRV at different stages was monitored by connecting electrocardiograms 5 min at baseline, 30 min during the intervention period, and 30 min after intervention. The mean values of six time segments of 5 min each during the intervention period and within 30 min after the intervention were compared with the baseline segment of 5 min to analyze the changes in HR and HRV in each group before, during, and after intervention (Joukar and Sheibani, 2017; Figure 3).

### Data collection and analysis

The frequency domain indices were: LF, HF, LF/HF, TP, VLF, LF nu, and HF nu. Table 1 shows the basic conditions.

**TABLE 1:** Selected frequency-domain measures of HRV.

Variable	Units	Description	Frequency range	Physiological significance
Analysis of short-term recordings (5 min)				
TP	ms^2^	The variance of NN intervals over the temporal segment	≤ 0.4 Hz	Total HRV during measurement
VLF	ms^2^	Power in very low frequency range	≤ 0.04 Hz	Related to sympathetic nerve regulation
LF	ms^2^	Power in low frequency range	0.04–0.15 Hz	Sympathetic nervous system regulation
LF nu	n.u.	LF power in normalized units LF/(Total Power-VLF) × 100		Balance between the two branches of the autonomic nerve
HF	ms^2^	Power in high frequency range	0.15–0.4 Hz	Reactive vagus regulation
HF nu	n.u.	HF power in normalized units HF/(Total Power -VLF) × 100		Balance between the two branches of the autonomic nerve
LF/HF		Ratio LF [ms^2^]/HF[ms^2^]		Balance of vagus nerve and sympathetic nerve

Although there is some debate about the range of frequencies influenced by ANS modulation in HRV, it is well established that only the parasympathetic activity (vagal activity) would affect the HF (Li and Zheng, 2022), and the parasympathetic drive to the heart is highly influenced by respiratory oscillations, while sympathetic function modulates the power of low frequencies from HRV spectra (Silva et al., 2017). Some studies suggested that LF, when expressed in normalized units (LF nu), is a quantitative marker of sympathetic modulation. The controlled and balanced behavior of the two branches of the ANS is emphasized by the representation of LF nu and HF nu. The sympathovagal balance seems to be accurately reflected by the fractional distribution of power across the frequency axis. This balance can be assessed by the relation between LF and HF components in normalized units or by the LF/HF ratio (Malliani et al., 1994). TP mainly reflects autonomic nerve tone and is influenced by both sympathetic and parasympathetic activity. VLF may have multiple influences, including thermoregulatory and hormonal factors. VLF may represent sympathetic activity (Ye et al., 2019).

### Statistical analyses

All measurements are expressed as the mean ± SEM for normal and non-normal distributions, respectively. Data on variables explored at HR and HRV between the control group and the model group after modeling and the HR and HRV frequency domain values of each group during and after acupuncture were compared with that before acupuncture were analyzed using repeated measures ANOVA followed by a Bonferroni multiple comparison test or Kruskal-Wallis ANOVA followed by a Wilcoxon signed rank test. The significance level was *P* < 0.05.

## Results

### Optimization of iaES frequency

**FIGURE 4:**
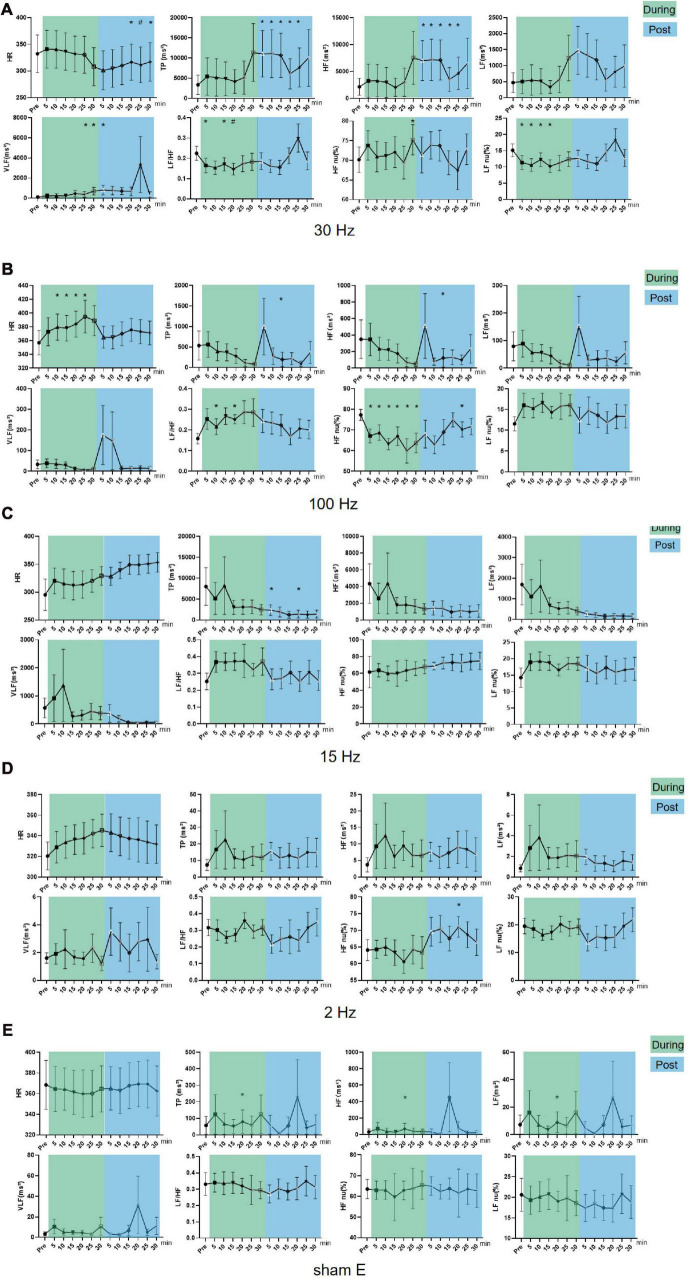
Effects of iaES at different frequencies on HR and HRV in normal rats. Rats were subjected to iaES immediately after their physiological state was stable, and the HR and HRV frequency domain values pre-, during- and post-intervention were recorded. The values of each group during and after acupuncture were compared with that before acupuncture by repeated measures variance test or non-parametric test (*n* = 8/group, *P < 0.05). Data are expressed as the mean ± standard deviation. **(A)** Frequency of iaES was 30 Hz. **(B)** Frequency of iaES was 100 Hz. **(C)** Frequency of iaES was 15 Hz. **(D)** Frequency of iaES was 2 Hz. **(E)** The iaES method was used without electroacupuncture (*P < 0.05, ^#^*P* < 0.01).

Of all the optional frequencies, 30 and 100 Hz iaES significantly changed HRV indicators. During intervention, 30 Hz iaES caused a decrease in LF nu and LF/HF (*P* < 0.05), and an increase in VLF and HF nu (*P* < 0.05). After the intervention, HF, VLF, and TP increased (*P* < 0.05), while HR decreased (*P* < 0.01; Figure 4A). These data indicate that the vagal activity, sympathetic activity, and the overall activity level of ANS clearly increased under 30 Hz iaES, with the ANS balance shifting to parasympathetic arm dominance.

With 100 Hz iaES, HR and LF/HF increased (*P* < 0.05) and HF nu decreased (*P* < 0.05) during intervention. After intervention, TP and HF decreased (*P* < 0.05; Figure 4B). Further, TP, LF, HF, and VLF showed decreasing trends during intervention. These data indicated that the 100 Hz iaES suppressed HRV in normal rats by larger amplitudes of vagal activity, shifting the ANS balance to sympathetic arm dominance and increasing HR.

Of the other groups, only TP, HF, and LF (*P* < 0.05) in the sham E group at 20 min during the intervention, HF nu (*P* < 0.05) in the 2 Hz group at 20 min after the intervention, and TP (*P* < 0.05) in the 15 Hz group at 5 min and 20 min after the intervention showed one-off positive results (Figures 4C–E).

Different frequencies of iaES drive specific regulatory directions. In normal rats, 30 Hz iaES increased vagal activity, promoted vagal dominance, and decreased HR. 100 Hz iaES increased HR by decreasing the proportion of vagal activity (Table 2). Thus 30 Hz and 100 Hz were considered the optimal parameters for use in subsequent experiments.

**TABLE 2:** Effects of iaES at 30 Hz and 100 Hz on HR and HRV in normal rats.

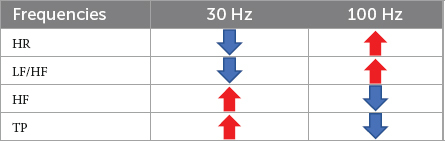

### Effect of optimized frequency iaES on ANS imbalance model rats

**FIGURE 5:**
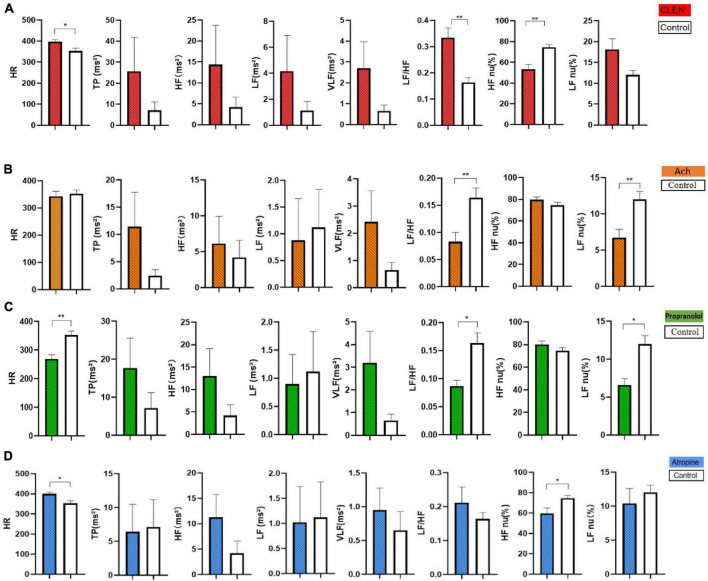
Comparison of HR and HRV of the control and the model groups after modeling. **(A)** The clenbuterol-treated rats were injected with 0.01% clenbuterol (0.5 mg/kg) intraperitoneally for 20 days consecutively. **(B)** The acetylcholine-treated rats were injected with 20% acetylcholine (0.1 mg/kg) for 7 days consecutively. **(C)** The propranolol-treated rats were given 0.2% propranolol (10 mg/kg) for 7 days consecutively. **(D)** The atropine-treated rats were injected with 0.1% atropine (4 mg/kg) for 7 days consecutively. Comparison of HR and HRV in the rats between the control group and the model group after modeling (*n* = 16/group). The data were analyzed by non-parametric test for five groups (control, CLEN, acetylcholine, propranolol, atropine), followed by a Dunnett’s test against a control group (Control is the normal group. *P < 0.05, **P < 0.01).

Sympathetic excited state model (clenbuterol-treated) rats exhibited lower HF nu (*P* < 0.01), and higher HR (*P* < 0.05), LF/HF (*P* < 0.01) than did normal rats. A higher LF/HF ratio is generally interpreted as an increase in sympathetic dominance or a decrease in parasympathetic dominance. LF is often associated with sympathetic activity, while HF is associated with parasympathetic activity. Because there is an upward trend in HF and LF, we excluded a decrease in parasympathetic dominance. Clenbuterol-treated rats reflecting a sympathetic excitation state (Figure 5A and Table 3).

**TABLE 3:** Comparison of HR and HRV of the control and the model groups after modeling.

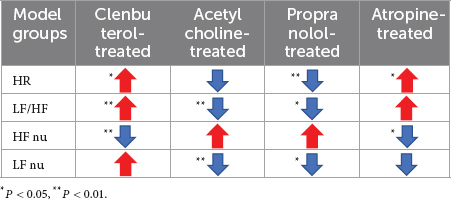

Parasympathetic excited state model (acetylcholine-treated) rats showed lower LF/HF and LF nu values than did normal rats (*P* < 0.01), reflecting a decrease in sympathetic dominance or an increase in parasympathetic dominance (Figure 5B). A lower LF/HF ratio is generally interpreted as a decrease in sympathetic dominance or an increase in parasympathetic dominance and LF is often associated with sympathetic activity, HF is associated with parasympathetic activity. HF shows an upward trend and LF indicates a downward trend. Thus, we believe that the acetylcholine-treated rats reflected a parasympathetic excitation state (Table 3). No significant increase in HF was observed, possibly because the half-life of acetylcholine is relatively short.

Sympathetic inhibited state models (Propranolol-treated) rats exhibited decreased HR (*P* < 0.01), LF nu (*P* < 0.05), and LF/HF (*P* < 0.05). A lower LF/HF ratio is generally interpreted as a decrease in sympathetic dominance or an increase in parasympathetic dominance. LF nu decreased, reflecting an inhibited sympathetic state (Figure 5C and Table 3).

Parasympathetic inhibited state model (atropine-treated) rats showed increased HR (*P* < 0.05) and decreased HF nu (*P* < 0.05). A higher LF/HF ratio is generally interpreted as an increase in sympathetic dominance or a decrease in parasympathetic dominance. HF nu decreased, reflecting an inhibited parasympathetic state (Figure 5D). Post-treated HR and HRV showed that chemical injections can be good models for ANS abnormalities (Table 3).

#### Effect of iaES on HRV in the sympathetic excited state rats

**FIGURE 6:**
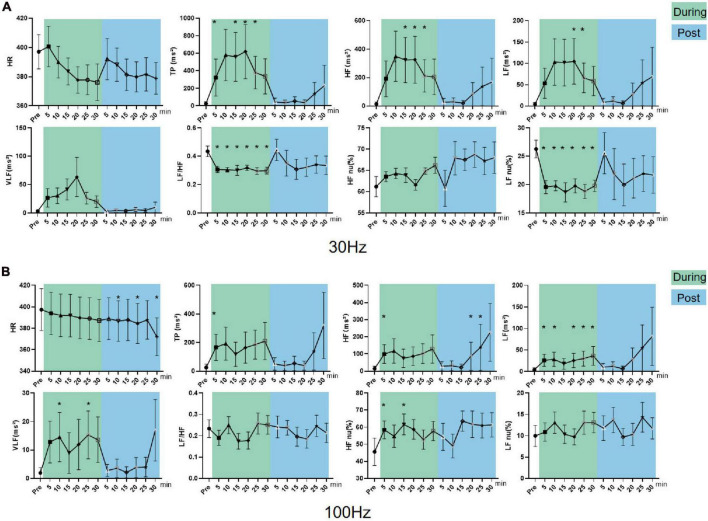
Effects of 30/100 Hz iaES on HRV and HR in sympathetic excited state rats. **(A)** 30 Hz iaES intervention was performed on the sympathetic excited state rats. **(B)** 100 Hz iaES intervention was performed on the sympathetic excited state rats. The frequency domain values of HRV were recorded for 30 min during and 30 min after the intervention. Data were analyzed by repeated measures variance test or non-parametric test (*n* = 8/group and the mean value of each phase in the middle and after the iaES was compared with that before the iaES) (*P < 0.05).

In sympathetic excited state rats, HF, LF, and TP increased (*P* < 0.05) and LF/HF and LF nu (*P* < 0.05) decreased significantly during 30 Hz iaES intervention (Figure 6A). These data indicated that 30 Hz iaES suppresses the sympathetic excited state by increase in vagal (HF) and sympathetic (LF) activity and overall autonomic level (TP) and decrease in the sympathetic arm (LF nu). A larger amplitude increment of vagal activity and the decrement of LF/HF and LF nu determined the ANS balance shifting toward parasympathetic arm dominance.

TP, HF, LF, VLF, and HF nu increased (*P* < 0.05) during 100 Hz iaES intervention. Further, HF increased (*P* < 0.05) and HR decreased (*P* < 0.05) after intervention (Figure 6B). Thus, 100 Hz iaES likely suppressed the sympathetic excited state by increasing vagal activity more substantially and increasing sympathetic activity and overall ANS activity. HF nu increases suggested a dominant increment of vagal activity.

30 Hz and 100 Hz iaES suppressed sympathetic excitation by increasing vagal activity and overall activity, re-establishing the balance by larger amplitude increments in vagal activity. 30 Hz iaES showed immediate effects on autonomic balance during intervention. 100 Hz iaES but not 30 Hz iaES caused lower HR after intervention, indicating a dominating inhibitory role for 100 Hz iaES on sympathetic excitation.

#### Effect of iaES on HRV in parasympathetic excited state rats

**FIGURE 7:**
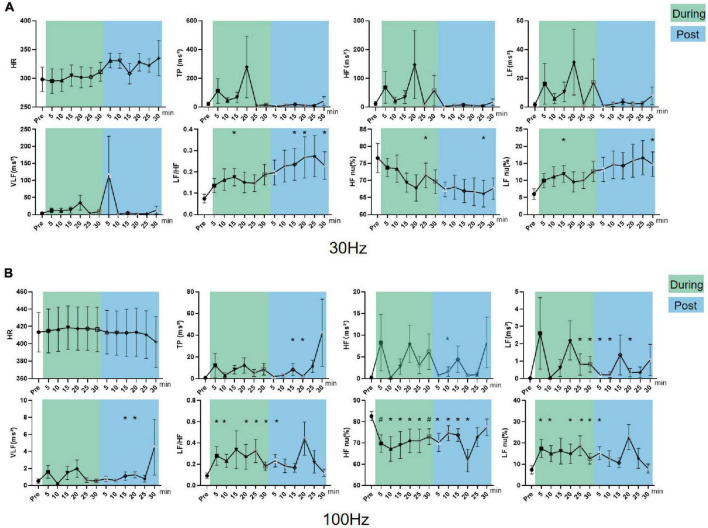
Effects of 30/100 Hz iaES on HRV and HR in parasympathetic excited state rats. **(A)** 30 Hz iaES intervention performed on the parasympathetic excited state rats. **(B)** 100 Hz iaES intervention performed on the parasympathetic excited state rats. The frequency domain values of HRV were recorded for 30 min during and 30 min after intervention. Data were analyzed by repeated measures variance test or non-parametric test (*n* = 8/group and the mean value of each phase in the middle and after the iaES was compared with that before the iaES) (*P < 0.05, ^#^*P* < 0.01).

In parasympathetic excited state rats, LF nu and LF/HF increased and HF nu decreased constantly during and after 30 Hz iaES (*P* < 0.05; Figure 7A). 30 Hz iaES suppressed the parasympathetic excited state by promoting larger amplitude increments on the sympathetic arm and relatively smaller amplitude increments in vagal activity, thereby rebalancing the ANS.

With 100 Hz iaES, LF/HF, LF nu, and LF increased (*P* < 0.05) while HF nu (*P* < 0.01) decreased during intervention. LF/HF (*P* < 0.05), LF nu (*P* < 0.05), and HF nu (*P* < 0.01) were altered immediately during intervention. LF/HF, LF nu, LF, VLF, HF, and TP increased and HF nu decreased after intervention (*P* < 0.05; Figure 7B). These data indicated that the 100 Hz iaES played a suppressive role in the parasympathetic excited state by larger amplitude increments of the sympathetic arm though vagal and the sympathetic increases.

For the parasympathetic excited state, 30 and 100 Hz iaES produced larger increments in sympathetic activity and relatively smaller increments in vagal activity, thereby restoring ANS balance. Notably, the 100 Hz intervention caused a predominant elevation of LF/HF and reduction of HF nu, indicating a stronger suppressive effect of 100 Hz iaES on the parasympathetic excitation state. Thus, 100 Hz iaES appears more beneficial for restoring balance in the excited state. 30 Hz iaES also exhibited a positive effect in restoring ANS balance.

#### Effect of iaES on HRV in sympathetic inhibited state rats

**FIGURE 8:**
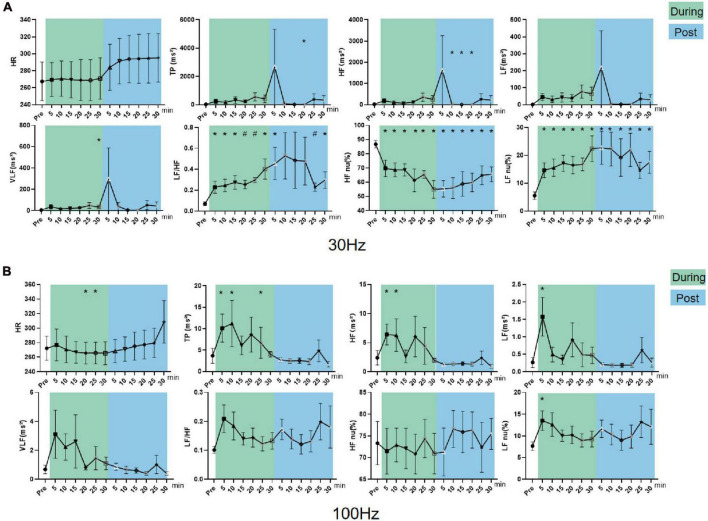
Effects of 30/100 Hz iaES on HRV and HR in sympathetic inhibited state rats. **(A)** 30 Hz iaES intervention was performed on sympathetic inhibited state rats. **(B)** 100 Hz iaES intervention was performed on sympathetic inhibited state rats. The frequency domain values of HRV were recorded for 30 min during and 30 min after the intervention. Data were analyzed by repeated measures variance test or non-parametric test (*n* = 8/group and the mean value of each phase in the middle and after the iaES was compared with that before the iaES) (*P < 0.05, ^#^*P* < 0.01).

In sympathetic inhibited state rats, LF/HF (*P* < 0.01), LF nu, and VLF (*P* < 0.05) increased and HF nu (*P* < 0.05) decreased during the intervention. LF/HF (*P* < 0.01) and LF nu (*P* < 0.05) increased while HF nu (*P* < 0.05) decreased after intervention (Figure 8A). These data indicated 30 Hz iaES could improve the sympathetic inhibited state by larger amplitude increments in sympathetic activity though sympathetic (VLF) and vagal activity (HF) increase.

With 100 Hz iaES, LF, HF, TP, and LF nu all increased (*P* < 0.05) while HR decreased (*P* < 0.05) during intervention (Figure 8B). These data indicate that 100 Hz iaES increases vagal, sympathetic, and overall activity. Complex interactions between the parasympathetic and sympathetic nervous systems have long been simplified to antagonism, emphasizing their opposition to each other. Our results showed an apparent increase in activity (medullary level) in the central sympathetic and parasympathetic nerves. The emergence of lower HR in the later phase of the intervention indicated an increase in vagal activity, decrease in sympathetic activity, or both. 100 Hz iaES applied to the sympathetic inhibited state rats suppressed the sympathetic arm and exacerbated the inhibited state. It could therefore be detrimental.

30 Hz iaES appeared suitable for intervention on the sympathetic inhibited state because of its ability to rebalance the ANS by larger amplitude increments in sympathetic and relatively smaller increments in vagal activity.

#### Effect of iaES on HRV in parasympathetic inhibited state rats

**FIGURE 9:**
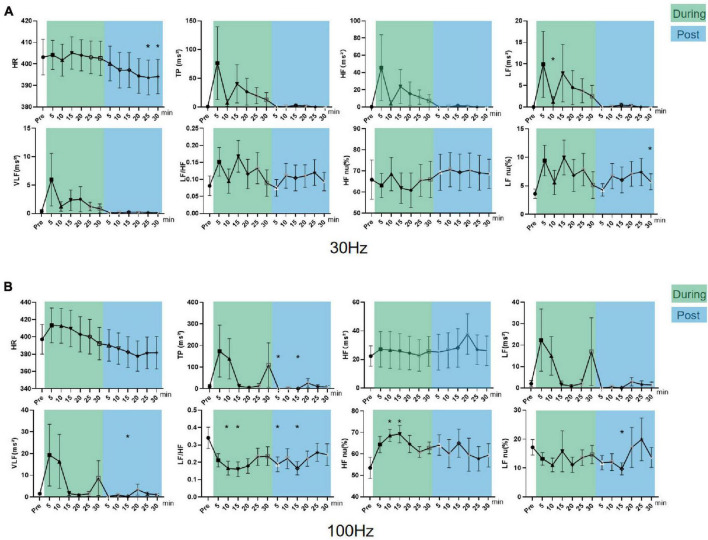
Effects of 30/100 Hz iaES on HRV and HR in parasympathetic inhibited state rats. **(A)** 30 Hz iaES intervention was performed on parasympathetic inhibited state rats. **(B)** 100 Hz iaES intervention was performed on parasympathetic inhibited state rats. The frequency domain values of HRV were recorded for 30 min during and 30 min after the intervention. Data were analyzed by repeated measures variance test or non-parametric test (*n* = 8/group and the mean value of each phase in the middle and after the iaES was compared with that before the iaES) (*P < 0.05).

In parasympathetic inhibited state rats, LF increased (*P* < 0.05) during 30 Hz iaES, and LF nu increased and HR declined (*P* < 0.05) after intervention (Figure 9A). The HR decrease indicated that 30 Hz iaES facilitates the vagal arm and ANS rebalance.

With 100 Hz iaES, HF nu increased and LF/HF decreased during intervention (*P* < 0.05). LF/HF and LF nu decreased after intervention (*P* < 0.05). These data indicated that the 100 Hz iaES shifted ANS balance toward the parasympathetic arm in a parasympathetic inhibited state (Figure 9B). The increase in HF nu and VLF indicated that both sympathetic activity and vagal activity increased with 100 Hz iaES, with a larger amplitude increment in vagal activity.

In the parasympathetic inhibited state, 30 Hz and 100 Hz iaES restored autonomic rebalance by larger increments in vagal activity and lower sympathetic activity. 30 Hz iaES intervention was particularly effective in reversing the elevated HR caused by atropine.

## Discussion

Frequency-dependency studies indicated that iaES has the advantage of more precise frequency control over manual acupuncture techniques. Of all the optional frequencies, 30 and 100 Hz iaES significantly changed HRV indicators compared with 2 and 15 Hz (Supplementary Figure 1). These findings are in agreement with Sclocco et al. (2020), where 100 Hz evoked stronger brainstem responses than 2 Hz. In addition, we believe that HF-HRV power was increased on average for 30 Hz stimulation, while the average HF-HRV power was decreased at 100 Hz. iaES-mediate HRV modulation is state- and frequency-dependent. Different states or frequencies have distinct regulation characteristics for peripheral outflow.

Primary state is an essential factor influencing HRV improvement with iaES. There are individual differences in the therapeutic effects of taVNS, which are related to ANS state (Wu et al., 2021; Jensen et al., 2022). ANS imbalance is involved in several diseases or disorders or could occur as a result of trauma, specific drug use, or exposure to toxins. ANS imbalance can manifest as a decrease in function (e.g., pure autonomic failure) or an increase in function (e.g., neurogenic hypertension) (Wehrwein et al., 2016). However, the parasympathetic excited state is usually manifested by relaxation and rest of the body, including lower heart rate (Malhotra et al., 2021), decreased blood pressure, increased digestive activity, slower breathing, and narrowed pupils of the eyes (Wehrwein et al., 2016). This state helps the body to recover and repair, and also helps to alleviate psychological stress or anxiety (dler-Neal et al., 2020; Magnon et al., 2021). Thus, the parasympathetic excited state differs from common pathologic states, appearing similar to the normal state, independent of autonomic imbalance pathological states.

100 Hz iaES suppresses the parasympathetic arm and increases the sympathetic arm in the non-pathological state, which is consistent with 100 Hz transcutaneous electrical nerve stimulation applied in the paravertebral ganglia (Stein et al., 2011). In common pathological states, 100 Hz iaES suppresses the sympathetic arm and increases the parasympathetic arm. 100 Hz iaES produces distinct effects in different states reflecting the state-dependency of iaES. State-dependent stimulation effects should be considered for altered ANS states. Considering the interaction between states and frequency factors may help to advance the treatment of ANS imbalance disorders with different iaES protocols, explain the strong variability in the observed effects, and promote the development of more effective individualized treatment.

Different frequencies of iaES drive distinct effects on HRV, particularly in restoring ANS homeostasis. In normal rats, 30 Hz iaES activates the parasympathetic nervous system (increase in HF and HF nu) and affects ANS balance by increasing vagal activity, with the best results in increasing overall HRV activity and reducing HR. 100 Hz iaES affects the ANS by decreasing vagal activity (decrease in HF and HF nu) to enhance the sympathetic arm, resulting in lower HRV and higher HR.

In the four imbalanced states designed in this study, the 30 Hz iaES exhibited a beneficial role on excitation or inhibition, while 100 Hz iaES appeared to suppress the sympathetic arm regardless of sympathetic excitation or sympathetic/parasympathetic inhibition (Supplementary Figure 2). 30 Hz iaES could therefore prove safe in clinical application. 100 Hz is the highest frequency available on commercially available EA apparatus and was therefore the upper frequency limit in this study. Alexander and McNamara (2012) demonstrated that short bursts of 100 Hz VNS elevated the seizure threshold in rats. However, very few studies have directly compared the effects of high and low frequencies on HRV.

iaES is a promising intervention for improving HRV and has produced effects similar to those of VNS in animal experiments. In an ANS imbalanced state, 30 Hz, iaES increases autonomic outflow, including sympathetic (increase in LF) and parasympathetic (increase in HF) activity, thereby promoting restoration of ANS equilibrium. The increase in the amplitude of the autonomic outflow is differentially distributed, with the weaker division receiving a greater increase than the stronger division, ultimately leading to a re-balance of sympathetic and parasympathetic outflow.

Our results suggest a central reciprocal pattern within the autonomic system. The improvement of HRV by iaES is likely accomplished by restoring autonomic stability through increased parasympathetic and sympathetic activities with varied amplitudes. Unlike usual strategies, which focus on either increasing parasympathetic activity or decreasing sympathetic activity, 30 Hz and 100 Hz iaES concurrently increases parasympathetic (increase in HF) and sympathetic activity (increase in LF) (Clancy et al., 2014).

Complex interactions between the parasympathetic and sympathetic nervous systems have been simplified to antagonism, emphasizing their opposition to each other (Arslan and Unal, 2022). Advances in neuro-immune interaction have highlighted atypical functional peripheral cooperation between the vagus and sympathetic signals in regulating innate immune response and inflammation (Bonaz, 2022). Our results showed an increase in activity (medullary level) in the central sympathetic and parasympathetic nerves during 30/100 Hz iaES. However, this was presented as a peripheral outflow after central integration. The individual sympathetic or parasympathetic activity detected peripherally should mainly be an undirected outcome after central balance (antagonism). Stimulation of the ABVN via the cymba concha activates the NST and LC. iaES enhances autonomic neurocentral integration by activating parasympathetic and sympathetic areas, as well as reaching afferent targets via the vagal pathway (Sclocco et al., 2020; Yap et al., 2020). This has the advantage of an “up-down” global regulatory effect, as ABVN acts as a central regulatory interface at the supra-spinal level (Badran et al., 2022a).

HRV is regarded as a noninvasive tool for assessing autonomic function (Gitler et al., 2022), and is essential to systematically examine how iaES affects ANS. HRV could be used to assess the impact of this intervention on autonomic function, and could potentially be utilized as a predictive biomarker of iaES responsiveness since it could be used to select the right individuals, stimulation sites, and stimulation dosage to optimize neuromodulation therapies (Soltani et al., 2023). Using this marker as a response evaluation tool in iaES could reflect response to treatment in real-time and allow optimization of patient selection and stimulation dosage.

In this study, ANS activity was monitored by HRV in anesthetized rats in order to reduce the interference from exercise and stress responses (Wu et al., 2019). Anesthetic drugs have a greater impact on ANS activity in animals, the choice of anesthetic drug was therefore made to minimize this influence. Urethane was used for HRV evaluation of cardiovascular autonomic nervous function in rats by Padley et al. (2005) and Harris et al. (2011). Urethane has a relatively mild inhibitory effect on the ANS, making it appropriate for studies of preserved autonomic reflex activity (Zhou and Qu, 2008). Thus, urethane was selected for animal anesthesia in this experiment.

This study has a few limitations. We observed the pattern of autonomic peripheral outflow effects after stimulation of afferent fibers of ABVN but could not explore the central mechanisms involved. Furthermore, only the 2 mA intensity was used, without investigating the effect of any intensity. 2 mA stimulation through these electrodes is similar to the amplitude used for human patients. Further, despite long-term pharmacological interventions, the quality of the modeling results was lower than expected owing to the use of intraperitoneal injections. The relatively short half-life of acetylcholine did not result in significant parasympathetic excitation. 30 Hz could improve HRV; 2 Hz and 15 Hz could not. This result correlated with the findings that the required parasympathetic peripheral electrical stimulation frequencies are 20–25 Hz (Kaniusas et al., 2019b; Cramer et al., 2023).

## Conclusion

iaES stimulation occurs via the ABVN, which is a peripheral shortcut access to act on ANS central and trigger ANS central integration, restore ANS central balance, and enhance vagal and sympathetic outflow. Moreover, iaES increases vagal activity and enhances overall autonomic activity and sympathetic activity and rebalances the ANS by greater amplitude increments on the relatively weaker side of sympathetic/parasympathetic nerves (proportion or activity).

iaES regulates ANS outflow in a pattern of state- and frequency-dependence (Table 4). 100 Hz iaES inhibits the parasympathetic arm and upregulates the sympathetic arm in normal and parasympathetic excited states. In the sympathetic excited/inhibited and parasympathetic inhibited states, 100 Hz iaES always inhibits the sympathetic arm. In a sympathetic inhibited state, risks may arise. 30 Hz iaES facilitates ANS in normal and imbalanced ANS rats.

**TABLE 4:** Effect of iaES on autonomic regulation.

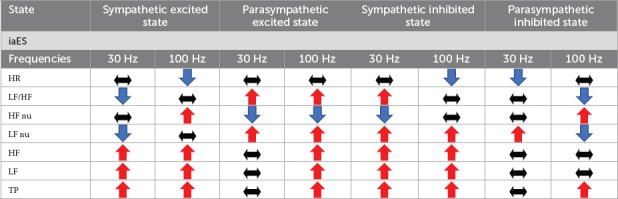

## Data availability statement

The raw data supporting the conclusions of this article will be made available by the authors, without undue reservation.

## Ethics statement

The animal study was approved by the Ethics Committee of the Animal Center of the Nanjing University of Chinese Medicine. The study was conducted in accordance with the local legislation and institutional requirements.

## Author contributions

SY: Conceptualization, Data curation, Investigation, Methodology, Software, Writing – original draft. Y-RW: Methodology, Resources, Supervision, Validation, Visualization, Writing – review & editing. ZZ: Methodology, Resources, Writing – original draft. Y-HP: Conceptualization, Formal analysis, Investigation, Project administration, Software, Writing – original draft. J-FJ: Conceptualization, Funding acquisition, Resources, Visualization, Writing – original draft, Writing – review & editing.
