# Supplementary material for: State- and frequency-dependence in autonomic rebalance mediated by intradermal auricular electroacupuncture stimulation
Source: Front Neurosci. 2024 May 23;18:1367266. doi: 10.3389/fnins.2024.1367266 (PMC11153749; doi:10.3389/fnins.2024.1367266)
Supplement: Supplementary file 1 [file Data_Sheet_1.docx]

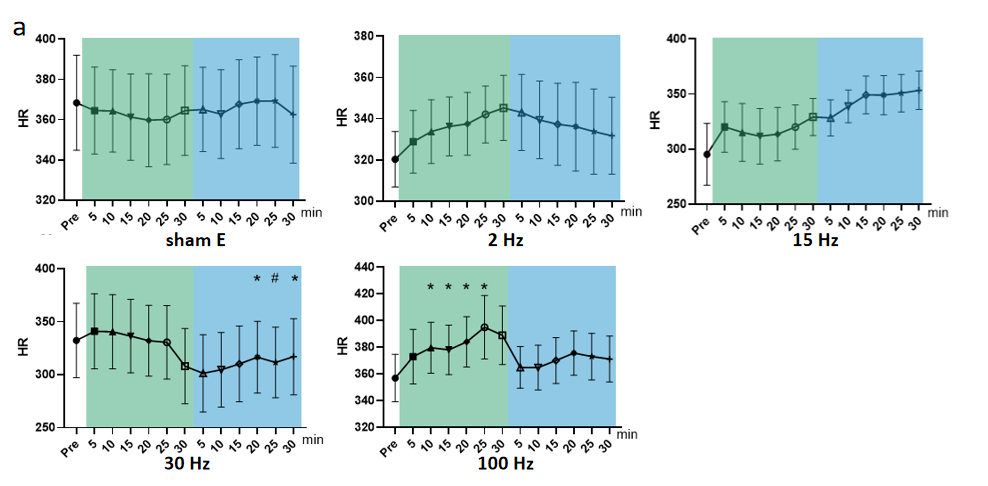

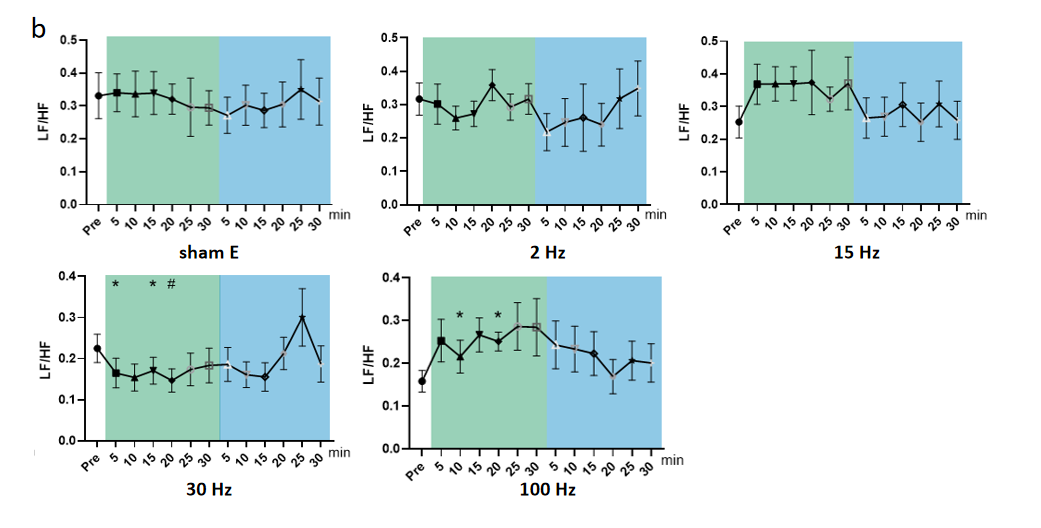


Supplementary Figure 1. Effects of iaES at different frequencies on HR and LF/HF-HRV in normal rats

Rats were subjected to iaES immediately after their physiological state was stable, and the HR and HRV frequency domain values pre-, during- and post-intervention were recorded. The values of each group during and after acupuncture were compared with that before acupuncture by repeated measures variance test or non-parametric test (n = 8/group, *P < 0.05). Data are expressed as the mean ± standard deviation. (a) Effects of iaES at different frequencies on HR in normal rats. (b) Effects of iaES at different frequencies on HR in normal rats. (Note: *P < 0.05, ^#^P < 0.01.)


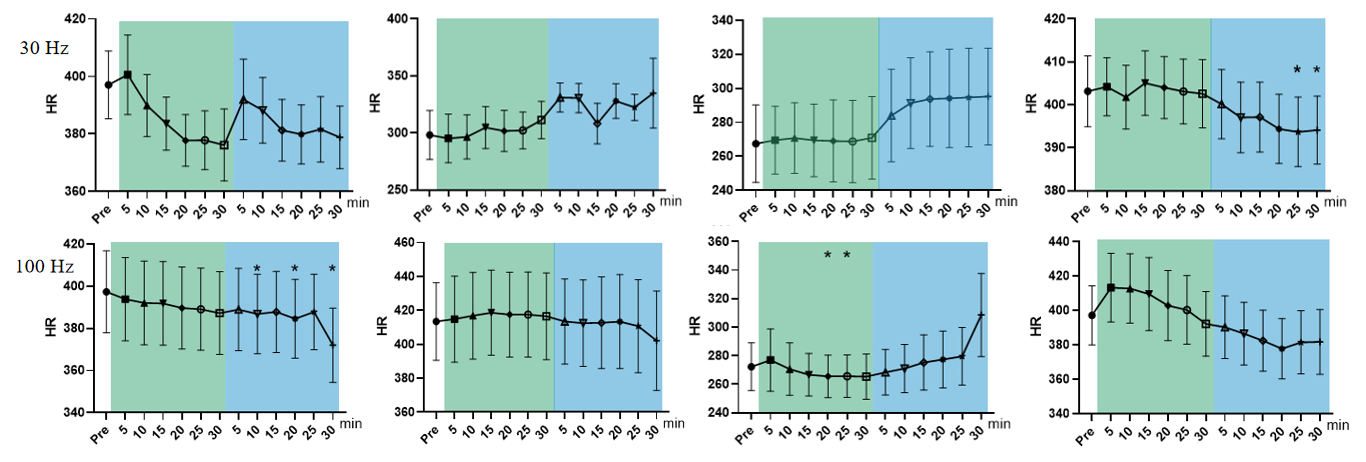

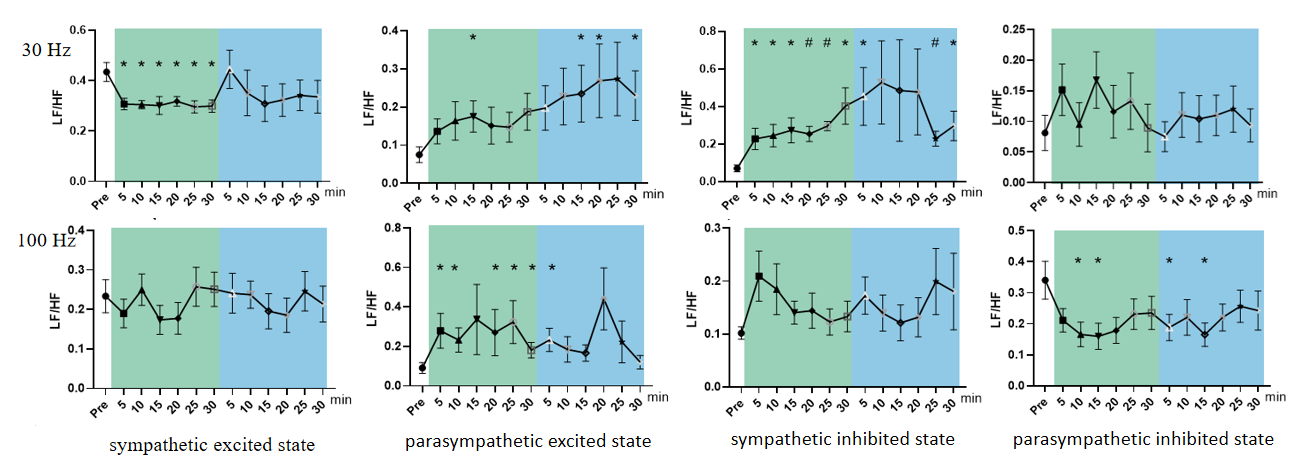


Supplementary Figure 2. Effect of 30/100 Hz iaES on ANS imbalance model rats

(a) 30 Hz iaES intervention performed on ANS imbalance model rats. (b) 100 Hz iaES intervention performed on ANS imbalance model rats. The frequency domain values of HRV were recorded for 30 min during and 30 min after intervention. Data were analyzed by repeated measures variance test or non-parametric test (n = 8/group and the mean value of each phase in the middle and after the iaES was compared with that before the iaES). (Note: *P < 0.05, ^#^P < 0.01)
